# Molecular Characterization and the Antimicrobial Resistance Profile of *Salmonella* spp. Isolated from Ready-to-Eat Foods in Ouagadougou, Burkina Faso

**DOI:** 10.1155/2022/9640828

**Published:** 2022-11-09

**Authors:** Adama Patrice Soubeiga, Dissinviel Stéphane Kpoda, Muller K. A. Compaoré, Asseto Somda-Belemlougri, Ndamiwe Kaseko, Sibiri Sylvain Rouamba, Sandrine Ouedraogo, Roukiatou Traoré, Paulette Karfo, Désiré Nezien, Fulbert Nikiéma, Elie Kabre, Cheikna Zongo, Aly Savadogo

**Affiliations:** ^1^Applied Biochemistry and Immunology Laboratory (LABIA), Sciences and Technologies Doctoral School, Joseph KI-ZERBO University, 03 BP 7021 03, Ouagadougou 03, Burkina Faso; ^2^National Public Health Laboratory, 09 BP 24, Ouagadougou 09, Burkina Faso; ^3^Ziniaré University Center, Joseph KI-ZERBO University, 03 BP 7021, Ouagadougou 03, Burkina Faso; ^4^Laboratory of Microbiology and Microbial Biotechnology (LAMBM), Sciences and Technologies Doctoral School, Joseph KI-ZERBO University, 03 BP 7021, Ouagadougou 03, Burkina Faso; ^5^Laboratory of Molecular Biology Epidemiology and Surveillance of Foodborne Agents (LaBESTA), Sciences and Technologies Doctoral School, Joseph KI-ZERBO University, 03 BP 7021, Ouagadougou 03, Burkina Faso; ^6^Kamuzu Central Hospital, Lilongwe, Malawi

## Abstract

The emergence of antimicrobial-resistantfood-borne bacteria is a great challenge to public health. This study was conducted to characterize and determine the resistance profile of Salmonella strains isolated from foods including sesames, ready-to-eat (RTE) salads, mango juices, and lettuce in Burkina Faso. One hundred and forty-eight biochemically identified Salmonella isolates were characterized by molecular amplification of Salmonella marker *invA* and *spiC*, *misL*, *orfL*, and *pipD* virulence genes. After that, all confirmed strains were examined for susceptibility to sixteen antimicrobials, and PCR amplifications were used to identify the following resistance genes: *bla*_TEM_, *temA*, *temB*, *StrA*, *aadA*, *sul1*, *sul2*, *tet(A)*, and *tet(B)*. One hundred and eight isolates were genetically confirmed as *Salmonella* spp. Virulence genes were observed in 57.4%, 55.6%, 49.1%, and 38% isolates for *pipD*, *SpiC*, *misL*, and *orfL*, respectively. Isolates have shown moderate resistance to gentamycin (26.8%), ampicillin (22.2%), cefoxitin (19.4%), and nalidixic acid (18.5%). All isolates were sensitive to six antibiotics, including cefotaxime, ceftazidime, aztreonam, imipenem, meropenem, and ciprofloxacin. Among the 66 isolates resistant to at least one antibiotic, 11 (16.7%) were multidrug resistant. The Multiple Antimicrobial Resistance (MAR) index of Salmonella serovars ranged from 0.06 to 0.53. PCR detected 7 resistance genes (*tet(A)*, *tet(B)*, *bla*_TEM_, *temB*, *sul1*, *sul2*, and *aadA*) in drug-resistant isolates. These findings raise serious concerns because ready-to-eat food in Burkina Faso could serve as a reservoir for spreading antimicrobial resistance genes worldwide.

## 1. Introduction

Food-borne diseases present a significant public health concern around the world. According to a recent report, 1 in 10 people become ill from consuming contaminated food, with 420,000 deaths yearly [1]. Among the diverse number of biological contaminants in existence, Salmonella is the most common pathogen responsible for thousands of deaths worldwide [2]. Salmonella is a Gram-negative, nonspore-forming bacillus, facultative anaerobe, and a member of the *Enterobacteriaceae* family. Based on differences in their 16S rRNA sequence analysis, the genus Salmonella is divided into two taxonomical species, including *Salmonella enterica* and *Salmonella bongori* [3]. Most isolates that cause diseases in humans and animals belong to *S. enterica*. This species is further divided into more than 2,600 serovars capable of causing illnesses in humans and animals [4]. Their reservoir is the gastrointestinal tract of a wide range of domestic and wild animals, with the ability to grow and multiply under various environmental conditions outside the living host. Salmonellosis occurs after the consumption of food or water that has been contaminated by Salmonella [5].

In humans, *S. enterica* serovars Typhi and Paratyphi cause severe systemic typhoid fever [6]. In contrast, nontyphoidal Salmonella (NTS), such as *Salmonella ser. Typhimurium* and *Salmonella ser. Enteritidis*, are commonly associated with gastroenteritis, the most common clinical presentation of Salmonella infections [6, 7]. The worldwide incidence of NTS is considerable, with an estimated 1.3 billion acute cases of gastroenteritis annually leading to 3 million deaths [8]. Africa has a high mortality rate, accounting for 4,100 deaths per year at a rate of 320 per 100,000 people [9]. The severity and outcome of the disease are associated with differences in host susceptibility, virulence factors, and serovar fitness [10]. The pathogenicity is mediated by virulence factors such as Salmonella pathogenicity islands (SPIs), plasmids, pili, and enterotoxins [10, 11]. SPIs, which are acquired through horizontal gene transfer events, carry the majority of the virulence genes of *Salmonella* [12].

In general, many cases of salmonellosis result in a self-limiting process in humans [6]. However, severe invasive disease or prolonged illness may require antimicrobial therapy, especially in children and immunocompromised [6]. Hence, antimicrobial resistance (AMR) in food-borne pathogens such as Salmonella severely threatens global public health. Currently, the annual death toll from AMR is estimated to be 700,000; by 2050, this situation is expected to rise to 10 million, with an economic loss of $100 trillion [13]. AMR may emerge in bacteria during the evolutionary process as cells accumulate spontaneous genetic mutations (resistance-mediating) in preexisting genes and then transfer them through vertical gene transfer [14]. However, the selective pressure created by antimicrobial agent abuse and overuse in humans and animals has accelerated the phenomenon [15]. Of particular concern is the widespread emergence of multidrug resistance (MDR) in Salmonella to clinically critical antimicrobial agents such as fluoroquinolones and *β*-lactams that could present a severe problem in human salmonellosis treatment [16, 17]. Contamination of food with MDR Salmonella is a significant public health problem, as food can be a vehicle for spreading and distributing antimicrobial-resistant strains to humans either by direct contact or indirectly by consuming contaminated foods [18]. Indeed, by consumption of food containing antibiotic-resistant bacteria, exchange between bacteria from food and human intestinal microorganisms could occur, leading to the accumulation of antibiotic-resistant genes in humans, which may affect the efficacy of antibiotics [19]. In developing countries such as Burkina Faso, the role of food chains in the emergence, selection, dissemination, and ultimately transmission of AMR has received much less attention. As a result, little scientific data on the antimicrobial resistance profiles of Salmonella serotypes in food matrices are available. The main objective of this study was to primarily characterize and determine the antibiotic resistance and virulence profiles of Salmonella isolated from foods received at the Laboratoire National de Santé Publique (LNSP) of Burkina Faso. The results could lay the foundation for further research on public health security and food safety problems caused by Salmonella.

## 2. Materials and Methods

### 2.1. Bacterial Collection

The study was conducted at the Direction du Contrôle des Aliments et de la Nutrition Appliquée of LNSP, the reference center for food quality control and applied nutrition in Burkina Faso. The studied strains were obtained through routine food surveillance activities from 2018 to 2020. The studied strains were obtained through routine food surveillance activities from January 2018 to December 2019. Four different food categories (sesames, *n* = 480; mango juices *n* = 156, lettuces, *n* = 92, and RTE salads, *n* = 324) comprising 1052 samples were analyzed during this period (Table 1).

The routine isolation and identification of *Salmonella* in this laboratory are performed according to the International Organization for Standardization procedure [20]. In brief, food samples (25 g or 25 mL) were preenriched in 225 mL of buffered peptone water (Liofilchem, Italy) and incubated at 37°C for 24 hours. One milliliter aliquots of the preenriched cultures were transferred to 10 mL of selenite cystine (Liofilchem, Italy). After incubation at 42°C for 24 hours, one loopful of each broth was streaked onto selective agar plates; xylose lysine deoxycholate agar (XLD, Liofilchem, Italy). The XLD plates were incubated at 37°C for about 24 hours. After incubation, the plates were examined for morphologically typical Salmonella colonies. Suspected Salmonella colonies were picked from each plate and subjected to biochemical tests using API20E Biochemical Test Strip (BioMérieux, France). Strains were isolated from sesames, ready-to-eat (RTE) salads, mango juices, and lettuce. All isolates were kept at −80°C in the brain heart infusion or BHI (Liofilchem, Italy) medium with 30% (v/v) glycerol until further testing.

### 2.2. DNA Extraction

All Salmonella isolates previously preserved were cultured on nutrient agar (micro master, India) at 37°C for 18–24 hours. The boiling method was utilized for each strain to extract the total DNA as defined earlier [21]. In brief, one loop full of Salmonella from the nutrient agar plates was subcultured overnight in 1000 *µ*L of BHI. The following day, each culture broth was centrifuged at 8,000*g* for 5 minutes at 4°C, and the cell pellets obtained were resuspended in 500 *µ*L of sterile distilled water and boiled at 100°C for 10 minutes in a water bath. The cell suspension was frozen at −20°C for 10 minutes and centrifuged at 8,000*g* for 5 minutes at 4°C. The supernatant was transferred into a fresh Eppendorf tube and stored at −20°C until use. DNA concentration and quality were assessed using a Jenway 737501 Genova Nano Micro-Spectrophotometer (Jenway, United Kingdom).

### 2.3. Molecular Confirmation of Salmonella

Biochemically identified Salmonella isolates were subjected to confirmation by polymerase chain reaction (PCR) using the *invA* gene, which contains unique sequences specific to the genus Salmonella and has been proven to be an essential target for their detection [22]. Furthermore, *iroB* gene amplification was used to detect *S. enterica* isolates. PCR reactions were performed in a reaction mixture comprising 4 *µ*L FIREPol® Master Mix (Solis BioDyne, Estonia), 0.4 *µ*L of each primer, 4 *µ*L of DNA template, and nuclease-free water to make a volume up to 20 *µ*L. The primers, the size in base pairs of the respective amplification products, and the references used for the detection of targeted genes are presented in Table 1. Aliquots (5 *μ*L) of amplification products were analyzed on 1.5% agarose gel by electrophoresis in 0.5X TBE buffer (0.1 M Tris, 0.1 M boric acid, and 0.002 M NaEDTA) and stained with ethidium bromide. A 100-bp DNA ladder (Thermo Scientific, USA) was used as a molecular size standard and visualized by UV light illumination. A negative control without template DNA was included in all experiments.

### 2.4. Detection of Virulence Genes

All confirmed Salmonella isolates were screened for the presence of virulent genes (*spiC, misL, orfL,*and *pipD*). PCR conditions, primers, the size in base pairs of the respective amplification products, and the references used for the detection of targeted genes are presented in Table 2.

### 2.5. Antimicrobial Resistance Testing

The Kirby–Bauer agar disk diffusion method was used to evaluate antimicrobial susceptibility to a panel of 16 antimicrobial agents. These tested antimicrobials were amoxicillin/clavulanic acid (AMC, 30 *μ*g), ampicillin (AMP, 10 *μ*g), cefoxitin (FOX, 30 *μ*g), cefepime (FEP, 30 *μ*g), cefotaxime (CTX, 30 *μ*g), ceftazidime (CAZ, 30 *μ*g), aztreonam (ATM, 30 *µ*g), chloramphenicol (C, 30 *μ*g), trimethoprim/sulfamethoxazole (STX, 1.25/23.75 *μ*g), nalidixic acid (NA, 30 *μ*g), tetracycline (TE, 30 *μ*g), imipenem (IMP, 10 *μ*g), meropenem (MEM, 10 *µ*g), gentamycin (CN, 10 *μ*g), amikacin (AK, 30 *µ*g), and ciprofloxacin (CIP, 10 *μ*g). They were selected based on their clinical or epidemiological significance to human and animal health. *Escherichia coli* (ATCC 25922) was used as a reference strain for quality control. After testing, the interpretation of the zones of inhibition was in accordance with the CLSI guidelines. Resistance patterns were determined by taking into account any nonsusceptible (*R* + *I*) phenotype. A strain was classified as MRD if it is resistant to at least one antimicrobial in 3 different antimicrobial classes. The Multiple Antimicrobial Resistance (MAR) index was determined according to the method of Krumperman [27] using the formula: *a*/*b*, where “*a*” is the number of antibiotics to which the particular isolate was resistant, and “*b*” is the number of antibiotics to which the particular isolate was exposed.

### 2.6. Antimicrobial Resistance Gene Screening

Genes with reported contributions to antimicrobial resistance were tested in this study. Thus, genes encoding resistance to ampicillin, *bla*_TEM_*, temA,* et, *temB*), tetracycline (*tet(A)*, et, *tet(B)*), sulfonamides (*sul1* and *sul2*), and gentamycin (*StrA* and *aadA*), selected according to the resistance phenotypes, were screened by PCR. The primers, the size in base pairs of the respective amplification products, and the references used for detection of targeted genes are presented in Table 2.

### 2.7. Statistical Analysis

The data were statistically analyzed using IBM SPSS 25.0 software. Chi-square (*χ*^2^) was used to determine if there was any significant difference between the prevalence of each virulence gene in Salmonella from mango juice, sesame, RTE salad, and lettuce. *P* values ˂0.05 were considered statistically significant.

## 3. Results and Discussion

Antimicrobial-resistant Salmonella in RTE foods is a big concern. As a result, regular monitoring of the prevalence and antibiotic resistance characteristics of Salmonella strains isolated from foods can provide epidemiological information about food-borne infection in a specific area. We characterized and then determined the antibiotic resistance profiles and resistance genes of *Salmonella* spp. strains isolated from RTE foods received at the LNSP of Burkina Faso.

### 3.1. Presumptive Salmonella Isolates and Molecular Confirmations

**Figure 1:**
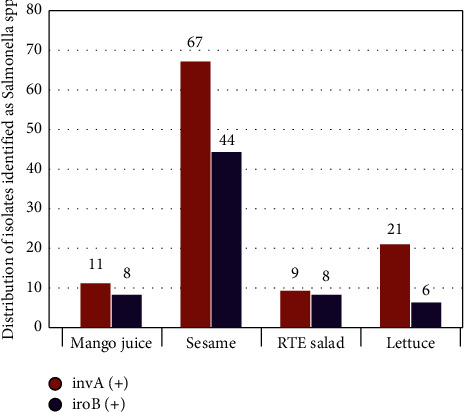
Number of confirmed Salmonella and *Salmonella enterica* detected in mango juices, sesames, RTE salads, and lettuces received at LNSP. *invA* (+) = PCR confirmed isolates and *IroB* (+) = *Salmonella enterica*.

From the 1052 samples analyzed during the study period, 148 were found as presumptive Salmonella isolates after cultural, biochemical identifications (Table 1). Presumptive Salmonella strains were isolated from sesame, RTE salad, lettuce, and mango juice samples. In the present study, we conducted PCR amplification using *invA* gene to confirm presumptive Salmonella strains isolated from sesame, RTE salad, lettuce, and mango juice samples. Out of the 148 biochemically identified isolates obtained from LNSP, only 108 were genetically confirmed as Salmonella through PCR amplification of the *invA* marker. This marker was amplified in 67, 21, 11, and 9 isolates from sesames, lettuce, mango juices, and RTE salads, respectively (Figure 1). Rahn et al. [22] demonstrated that the *invA* gene contains Salmonella-specific sequences and could be used as a PCR target with potential diagnostic applications. Furthermore, Salmonella serovars' *invA* gene enables bacteria to penetrate the host and induce infection, enhancing the pathogenicity of isolates [28]. Interestingly, we have noticed that the number of Salmonella is decreased after molecular confirmation demonstrating the sensitivity and reliability of the molecular amplification tests than traditional cultural methods. In addition to *invA* gene detection, we used the PCR assay with primers targeting the *iroB* gene. The PCR detection assay targeting *iroB* distinguishes *S. enterica* strains from other bacterial species, including *S. bongori* [29]. Out of 108 Salmonella isolates, 66 (61.1%) were identified as *S. enterica.* Most isolates that cause diseases in humans and animals belong to *S. enterica*. In developing countries such as Burkina Faso, numerous studies have previously reported the presence of *Salmonella* spp. in RTE foods [30–32]. The potential hygiene breakdowns at various stages of the food processing and distribution chain due to the lack of food safety regulations and food handler educations are responsible for food contamination in developing countries [33]. As the presence of pathogens such as Salmonella could cause illnesses with substantial health risks, including the death of consumers, routine monitoring is necessary to be undertaken.

### 3.2. Virulence Genes among Salmonella Isolates

**Figure 2:**
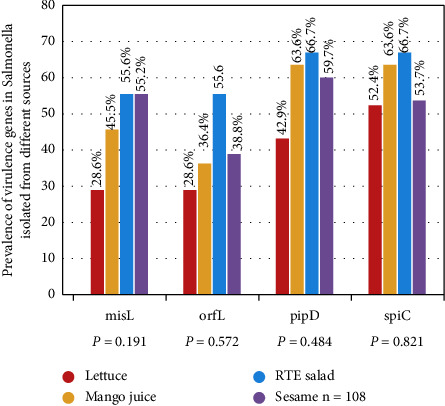
Prevalence of virulence genes in Salmonella isolates from sesame, RTE salad, mango juices, and lettuce samples.

Virulence determinants influence bacterial pathogenicity, and their presence in Salmonella can result in infections. During contamination, the host infection outcomes are shaped by a range of factors, including age, environment, and pathogenicity of the pathogen [26]. The present study established the prevalence of four virulence genes, including *spiC, pipD, misL, and orfL,* located on SPI. The SPIs contain a number of functionally related genes necessary for a specific virulence phenotype of Salmonella [34]. In this study, 57.4% of Salmonella isolates harbored the pipD gene, while 55.6%, 49.1%, and 38% were positive for *spiC*, *misL*, and *orfL*, respectively. High prevalence rates of *misL* and *pipD* (91% each) among Salmonella isolates from human diarrhea, environment, and lettuce samples in Burkina Faso have been reported [35]. In addition, Zishiri et al. [26] have found high detection (85%) of the *spiC* gene, followed by *pipD* (80%), *misL* (75%), and *orfL* (20%) among Salmonella isolated from human clinical samples in South Africa. Furthermore, Dione et al. [36] reported high prevalence rates of *pipD* (95%) and *spiC* (78%) in Salmonella isolated from humans and livestock samples. In contrast, low prevalence rates of 47% for *SpiC* and 35% for *pipD* in *Salmonella* spp. isolated from chickens in South Africa were reported [26]. The frequency of virulence genes in Salmonella isolates from lettuce, mango juice, RTE salad, and sesame are shown in Figure 2. After statistical analysis, there was no significant difference between the prevalence of each virulence gene in Salmonella from mango juice, sesame, RTE salad, and lettuce. Because Salmonella pathogenicity is determined by the presence or absence of virulence genes, the detection of different virulence genes in the Salmonella isolates in this study poses a potential public health risk.

### 3.3. Antimicrobial Resistance Profiles of *Salmonella* spp. Isolates

Antimicrobial resistance in Salmonella is rapidly becoming a major concern for both animals and humans globally [37]. RTE foods have been recognized as AMR bacteria reservoirs, directly threatening human health [24]. As a result, determining Salmonella antimicrobial resistance is essential for the treatment regimens during epidemics in a given location. In the present study, antimicrobial sensitivity testing using the disc diffusion method was performed on 108 Salmonella isolates recovered from food samples with 16 antimicrobial agents. Globally, 66 (61.1%) isolates showed resistance to at least one antimicrobial. The distribution of the susceptibility profile of Salmonella is presented in Table 3. Resistance to gentamycin (26.8%), ampicillin (22.2%), cefoxitin (19.4%), and nalidixic acid (18.5%) was the most common. Ampicillin, chloramphenicol, and trimethoprim-sulphamethoxazole constitute the first-line traditional antibiotics used for salmonellosis treatment [38]. High prevalence rates of resistance to these antibiotics have been described in Burkina Faso and elsewhere from other sources (e.g., humans and animals) [23, 36, 39]. Salmonella resistance to these affordable antimicrobials is a public health threat. Over the years, the misuse and abuse of these inexpensive and easily accessible over-the-counter drugs rendered them ineffective [40]. Interestingly, no Salmonella isolate was resistant to the third-generation cephalosporins (cefepime, cefotaxime, and ceftazidime). This is important for public health since these antimicrobials may be essential in threatening drug-resistant Salmonella pathogens. By contrast, Karikari et al. [40] in Ghana have reported high resistance of Salmonella isolated from salad to third-generation cephalosporins including cefotaxime (70%), ceftriaxone (85%), and ceftazidime (70%). In other countries, resistance against these antibiotics in Salmonella was highest, especially in poultry and pork [37, 41]. The high resistance to these antimicrobials results from the extensive use of these antibiotics for growth promotion and therapeutic and prophylactic purposes in livestock breeding in these areas [14]. Therefore, educating people about not to misuse these precious antimicrobials is essential to preserve their efficacy. In this study, Salmonella isolates showed moderate (18.5%) resistance to nalidixic acid (first-generation quinolones), which requires continuous monitoring since nalidixic acid resistance usually increases the risk of ciprofloxacin (second-generation quinolones) and other quinolone resistances [42].

The antimicrobial resistance profile and MAR index of the Salmonella serovars are presented in Table 4. The MAR index ranged from 0.06 (resistant to one antibiotic) to 0.53. A total of 10 (9.3%) of Salmonella were resistant to 2 antibiotics (AMP and FOX) which belonged to 2 different antimicrobial groups with a MAR index of 0.13. Furthermore, 1 (0.9%) Salmonella isolated from sesame was resistant to 8 antibiotics (AUG, AMP, FOX, TE, NA, CN, SXT, and *C*) with the MAR index of 0.53. Salmonella strains with a MAR index >0.2 originated from an environment where antimicrobials are overused and misused [27]. A total of 11 (10.2%) of Salmonella isolates were MDR bacteria (exhibited resistance to three or more classes of antimicrobial agents). The number of MDR isolates found in the current study was low compared to other studies from different countries [43–45]. Nikiema et al. [46] also found a similar moderate percentage of MDR strains Salmonella strains isolated from sandwiches sampled at street food establishments in Ouagadougou, Burkina Faso. By contrast, a relatively high prevalence of MDR Salmonella (35.97%) from chicken and guinea fowl in Burkina Faso has been reported by Caroline Bouda et al. [47]. The low number of MDR Salmonella isolates in this investigation can be attributed to the fact that the isolates were from samples that did not contain meat or fish. MDR strains in foods directly threaten human health as they could provoke diseases challenging to cure. In addition, a genetic element such as integrons is often associated with multiresistant phenotypes among Salmonella isolates and plays an essential role in spreading antimicrobial resistance genes among Gram-negative bacteria [48].

### 3.4. Antimicrobial Resistance Genes Detected

Table 5 shows the distribution of resistance genes among phenotypically resistant Salmonella isolates. PCR detected 8 resistance genes (*tet(A), tet(B), bla*_TEM_*, temB, sul1, sul2,* and *aadA*) in drug-resistant isolates. Salmonella develops AMR by complex mechanisms, such as the production of enzymes that can alter the antibiotic target site by activating efflux pumps and the production of *β*-lactamases, which can degrade the structure of antibiotic molecules [49]. Most AMR genes are on integrons, plasmids, or transposons, which may be transferred and mobilized to other or the same species of bacteria [48]. In the present study, 2 of the 5 tetracycline-resistant isolates harbored the *tet(A)* gene (40%), and only one isolate was positive for the *tet(B)* gene (20%). The genes *tet(A)* and *tet(B)* encode energy-dependent efflux proteins that help bacteria eliminate tetracycline from their cells [50]. These genes are widespread among Salmonella and have been located on transferable plasmids and code for an energy-dependent efflux pump system [23]. In the present study, one of the 4 sulfamethoxazole-resistant isolates carried the *sul1* gene (25%), 2 carried the *sul2* gene (50%), and one carried both the *sul1* and *sul2* genes (25%). These genes encode dihydropteroate synthase and are often found in integron-positive isolates that carry other genes [51]. The resistance of Gram-negative bacteria to cephalosporins and ampicillin is mainly mediated by *β*-lactamases [44]. Although TEM *β*-lactamase genes are nonextended-spectrum*β*-lactamase lactamases, *bla*_TEM_ is a major mediator of *β*-lactam resistance in *Salmonella* spp. worldwide [52]. In this study, 31 isolates resistant to ampicillin or cefoxitin were tested for three types of beta-lactamase genes. Among them, 15 (48.4%) gave positive amplicons for *bla*_TEM_, while 5 (20.8%) isolates exhibited the *temB* gene, and none of the *temA* genes were found. Similar to our findings, high rates of *bla*_TEM_ (44.11%) were found by Rajaei et al. [53] in *Salmonella* spp. isolates from raw kebab and hamburger in Iran and by Moawad et al. [54] in Egypt (73.3%) in *Salmonella* spp. isolates from chicken and beef meat. Aminoglycoside resistance occurs through several mechanisms that can coexist simultaneously in the same cell, the most common being chemically modifying aminoglycosides by aminoglycoside-modifying enzymes [55]. Enzymatic inactivation by acetylation, adenylylation, or phosphorylation at different locations of the aminoglycoside molecule is among the most clinically relevant strategies that bacteria use to resist the action of these antibiotics [56]. Aminoglycoside nucleotidyl-transferases can confer resistance to gentamicin, tobramycin, or streptomycin including aad and variant StrA/B among Gram-negative bacteria [57]. In our study, the *aadA* genes were found in high frequency (86.2%), while no isolate was found harboring *StrA.*

## 4. Conclusion

The study provides additional information about the presence of Salmonella in foods, especially RTE foods in Burkina Faso. Moreover, these might act as reservoirs for antimicrobial-resistant Salmonella, which constitute a risk to consumers. Thus, our findings highlight the urgent need for stringent sanitation and hygienic standards for food safety measures to minimize cross-contamination during the food chain process. In addition, it is crucial to implement strict guidelines for antimicrobial usage in hospitals and community settings to limit the potential spread of bacterial resistance.

## Figures and Tables

**Table 1:** Samples and isolates from different food sources.

Products	No. of samples	No. of samples positive for *Salmonella* spp.	Positive rate (%)
Sesame	480	71	14.8
Mango juice	156	18	11.5
RTE salad	324	21	6.5
Lettuce	92	38	41.3

**Table 2:** Primer sequences, PRC conditions, and the source of primers for amplification of antimicrobial resistance genes used for the study.

Target genes		Primer sequence (5′ to 3′)	PCR conditions	Product size	Reference
*bla *_TEM_	*F*	ACCAATGCTTAATCAGTGAG	3 min at 94°C; 35 cycles of 1 min at 94 C, 1 min. at 50°C and 1 min at 72°C; 10 min at 72°C	857 bp	[23]
	*R*	ACCAATGCTTAATCAGTGAG			
*temA*	*F*	ATGAGTATTCAACATTTCCG	3 min at 94°C; 30 cycles of 30 s at 94°C, 30 s at 62°C and 1 min at 72°C; 7 min at 72°C	867 bp	[23]
	*R*	CTGACAGTTACCAATGCTTA			
*temB*	*F*	TTTTCGTGTCGCCCTTATTCC	3 min at 94°C; 30 cycles of 30 s at 94°C, 30 s at 62°C and 1 min at 72°C; 7 min at 72°C	798 bp	[23]
	*R*	CGTTCATCCATAGTTGCCTGACTC			
*tet(A)*	*F*	GTGAAACCCAACATACCCC	5 min at 94°C; 30 cycles of 30 s at 94°C, 30 s at 60°C and 30 s at 72°C; 7 min at 72°C	888 bp	[24]
	*R*	GAAGGCAAGCAGGATGTAG			
*tet(B)*	*F*	CCTTATCATGCCAGTCTTGC	5 min at 94°C; 30 cycles of 30 s at 94°C, 30 s at 60°C and 30 s at 72°C; 7 min at 72°C	774 bp	[24]
	*R*	ACTGCCGTTTTTTCGCC			
*Sul1*	*F*	TCACCGAGGACTCCTTCTTC	3 min at 94°C; 30 cycles of 30 s at 94°C, 30 s at 53°C and 1 min at 72°C; 7 min at 72°C	435 bp	[24]
	*R*	CAGTCCGCCTCAGCAATATC			
*Sul2*	*F*	GCGCTCAAGGCAGATGGCAT	3 min at 94°C; 30 cycles of 30 s at 94°C, 30 s at 53°C and 1 min at 72°C; 7 min at 72°C	293 bp	[24]
	*R*	GCGTTTGATACCGGCACCCT			
*StrA*	*F*	CCAATCGCAGATAGAAGGC	3 min at 94°C; 30 cycles of 30 s at 94°C, 30 s at 58°C and 1 min at 72°C; 7 min at 72°C	548 bp	[25]
	*R*	CTTGGTGATAACGGCAATTC			
*aadA*	*F*	GTGGATGGCGGCCTGAAGCC	3 min at 94°C; 30 cycles of 30 s at 94°C, 30 s at 58°C and 1 min at 72°C; 7 min at 72°C	528 bp	[25]
	*R*	AATGCCCAGTCGGCAGCG			
*invA*	*F*	TCATCGCACCGTCAAAGGAACC	30 s at 95°C; 34 cycles of 30 s at 95°C, 30 s at 58°C and 1 min at 72°C; 5 min at 72°C	284 bp	[26]
	*R*	GTGAAATTATCGCCACGTTCGGGCAA			
*iroB*	*F*	TGCGTATTCTGTTTGTCGGTCC	30 s at 95°C; 34 cycles of 30 s at 95°C, 40 s at 55°C and 1 min at 72°C; 5 min at 72°C	606 bp	[26]
	*R*	TACGTTCCCACCATTCTTCCC			
*spiC*	*F*	CCTGGATAATGACTATTGAT	12 min at 94°C; 34 cycles of 1 min at 94°C, 30 s at 54°C and 5 min at 72°C; 5 min à 72°C^*∗*^	309 bp	[26]
	*R*	AGTTTATGGTGATTGCGTAT			
*misL*	*F*	GTCGGCGAATGCCGCGAATA	3 min at 94°C; 35 cycles of 1 min at 94°C, 1 min·s at 58°C and 1 min at 72°C; 5 min at 72°C	400 bp	[26]
	*R*	GCGCTGTTAACGCTAATAGT			
*orfL*	*F*	GGAGTATCGATAAAGATGTT	3 min at 94°C; 35 cycles of 1 min at 94°C, 1 min·s at 58°C and 1 min at 72°C; 5 min at 72°C	550 bp	[26]
	*R*	GCGCGTAACGTCAGAATCAA			
*pipD*	*F*	CGGCGATTCATGACTTTGAT	5 min at 94°C; 34 cycles of 25 s at 94°C, 30 s at 56°C and 50 s at 72°C; 5 min at 72°C	350 bp	[26]
	*R*	CGTTATCATTCGGATCGTAA			

^*∗*^5 s were added to the extension time each cycle.

**Table 3:** Antimicrobial resistance of Salmonella serovars from sesames, mango juices, lettuces, and RTE salads.

Antimicrobials	Sesame (*n* = 67)	Mango juice (*n* = 11)	Lettuce (*n* = 21)	RTE salad (*n* = 9)	Total (*n* = 108)
	Resistant (%)	Resistant (%)	Resistant (%)	Resistant (%)	(%)
Ampicillin	15 (22.4)	1 (9.0)	5 (23.8)	3 (26.7)	24 (22.2)
Amoxicillin-clavulanate	4 (6.0)	0	5 (23.8)	0 (16.7)	9 (8.3)
Cefoxitin	13 (19.4)	1 (9.0)	3 (14.3)	4 (23.3)	21(19.4)
Cefotaxime	0 (0)	0 (0)	0 (0)	0 (0)	0 (0)
Ceftazidime	0 (0)	0 (0)	0 (0)	0 (0)	0 (0)
Aztreonam	0 (0)	0 (0)	0 (0)	0 (0)	0 (0)
Imipenem	0 (0)	0 (0)	0 (0)	0 (0)	0 (0)
Meropenem	0 (0)	0 (0)	0 (0)	0 (0)	0 (0)
Tetracycline	2 (3.0)	1 (9.0)	2 (9.5)	0 (6.7)	5 (4.6)
Gentamycin	26 (38.8)	0 (0)	1 (4.8)	2 (10.0)	29 (26.8)
Amikacin	3 (4.5)	1 (9.0)	0	0 (0.0)	4 (3.7)
Chloramphenicol	5 (7.5)	0 (0)	5 (23.8)	0 (3.3)	6 (5.5)
Trimethoprim-sulphamethoxazole	2 (3.0)	1 (9.0)	1 (4.8)	0 (3.3)	4 (3.7)
Nalidixic acid	12 (18.0)	3 (22.3)	2 (9.5)	3 (16.7)	20 (18.5)
Ciprofloxacin	0	0		0	0

**Table 4:** Antibiotic resistance profile of Salmonella isolates illustrating multiple drug resistance and multiple antibiotic resistance (MAR) indexes.

Antibiotic-resistantprofiles	No. of antimicrobials	No. of antimicrobial classes	No. of isolates	Sources	MAR index
NA, AK	2	2	1	Mango juice	0.13
NA, CN	2	2	1	Sesame	0.13
AMP, CN	2	2	1	Sesame	0.13
AMP, FOX	2	2	10	RTE salad, sesame	0.13
TE, SXT	2	2	1	Mango juice	0.13
CN, AK	2	1	1	Sesame	0.13
NA, *C*	2	2	1	RTE salad	0.13
AUG, AMP	2	2	1	Sesame	0.13
FOX, CN	2	2	1	Sesame	0.13
AMP, NA, SXT	3	3	1	Sesame	0.2
FOX, NA, CN	3	3	2	RTE salad, sesame	0.2
AMP, CN, AK	3	2	1	Sesame	0.2
AUG, AMP, CN	3	3	1	RTE salad	0.2
AUG, AMP, FOX	3	3	3	RTE salad	0.2
AUG, AMP, FOX, CN	4	4	1	Sesame	0.26
AUG, AMP, NA, CN, AK	5	4	1	Sesame	0.33
AUG, AMP, TE, NA, SXT, *C*	6	6	1	RTE salad	0.4
AUG, AMP, FOX, TE, NA, CN, SXT, *C*	8	8	1	Sesame	0.53

TE = tetracycline, NA = nalidixic acid, AK = amikacin, SXT = sulphamethoxazole-trimethoprim, AMP = ampicillin, AUG = amoxicillin + clavulanic acid, FOX = cefoxitin, CN = gentamicin, and C = chloramphenicol.

**Table 5:** Occurrence of resistance genes among Salmonella isolates.

Antimicrobial resistance	Number of resistant isolates	Genes detected	Number of isolates
Ampicillin	31	*temB*	5
Cefoxitin		*bla *_TEM_	15
Tetracycline	5	*tet(A)*	2
		*tet(B)*	1
Sulfamethoxazole	4	*Sul1*	1
		*Sul2*	2
Aminoglycoside	29	*StrA*	0
		*aadA*	25

## Data Availability

The data used to support the findings of this study are available from the corresponding authors upon request.
